# A novel link between chronic inflammation and humanin regulation in children

**DOI:** 10.3389/fendo.2023.1142310

**Published:** 2024-01-23

**Authors:** Yunhan Zhao, Outi Mäkitie, Saila Laakso, Vera Fedosova, Lars Sävendahl, Farasat Zaman

**Affiliations:** ^1^ Department of Women’s and Children’s Health, Karolinska Institutet and Pediatric Endocrinology Unit, Karolinska University Hospital, Solna, Sweden; ^2^ Children’s Hospital, Helsinki University Hospital, University of Helsinki, Helsinki, Finland; ^3^ Department of Molecular Medicine and Surgery, Karolinska Institutet, and Clinical Genetics, Karolinska University Hospital, Stockholm, Sweden

**Keywords:** IBD, TNF, growth plate, humanin, chondrocyte

## Abstract

**Objective:**

Children with inflammatory bowel disease (IBD) often suffer from poor bone growth and impaired bone health. Humanin is a cytoprotective factor expressed in bone and other tissues and we hypothesized that humanin levels are suppressed in conditions of chronic inflammation. To address this, humanin levels were analyzed in serum samples from IBD patients and in *ex vivo* cultured human growth plate tissue specimens exposed to IBD serum or TNF alone.

**Methods:**

Humanin levels were measured by ELISA in serum from 40 children with IBD and 40 age-matched healthy controls. Growth plate specimens obtained from children undergoing epiphysiodesis surgery were cultured *ex vivo* for 48 hours while being exposed to IBD serum or TNF alone. The growth plate samples were then processed for immunohistochemistry staining for humanin, PCNA, SOX9 and TRAF2 expression. Dose-response effect of TNF was studied in the human chondrocytic cell line HCS-2/8. *Ex vivo* cultured fetal rat metatarsal bones were used to investigate the therapeutic effect of humanin.

**Results:**

Serum humanin levels were significantly decreased in children with IBD compared to healthy controls. When human growth plate specimens were cultured with IBD serum, humanin expression was significantly suppressed in the growth plate cartilage. When cultured with TNF alone, the expression of humanin, PCNA, SOX9, and TRAF2 were all significantly decreased in the growth plate cartilage. Interestingly, treatment with the humanin analog HNG prevented TNF-induced bone growth impairment in cultured metatarsal bones.

**Conclusion:**

Our data showing suppressed serum humanin levels in IBD children with poor bone health provides the first evidence for a potential link between chronic inflammation and humanin regulation. Such a link is further supported by the novel finding that serum from IBD patients suppressed humanin expression in *ex vivo* cultured human growth plates.

## Introduction

Children with chronic inflammatory diseases, such as inflammatory bowel disease (IBD), often experience impaired bone health including bone growth retardation (1, 2). Although many of these children are effectively treated with glucocorticoids and other anti-inflammatory therapies, bone growth retardation often remains or even worsen due to undesired side-effects of the treatment. A better understandings of how chronic inflammatory disorders affect bone health may facilitate the development of new bone-protective treatment strategies.

Longitudinal bone growth occurs at the growth plate, a hyaline cartilage layer situated in the metaphysis of long bones. In the growth plate, chondrocytes undergo proliferation and hypertrophy while producing cartilage matrix. During these steps, new cartilage tissue is formed which is subsequently remodeled into trabecular bone (3). This process is tightly controlled by a variety of growth factors, cytokines and ubiquitin/proteasome system (4), and any disturbance of this will result in growth retardation. Inflammation is characterized by active immune cells and elevated production of cytokines including tumor necrosis factor (TNF) which has been associated with disease activity in IBD. Furthermore, TNF has been linked to growth retardation and anti-TNF treatment has been found to have the capacity to rescue bone growth in patients with IBD (5, 6). Experimental data have suggested that circulating TNF may impair the growth hormone–insulin-like growth factor-1 axis (7, 8). Locally at the growth plate level, TNF and IL-1beta have been shown to act in synergy suppressing chondrocyte proliferation and bone growth in *ex vivo* cultured metatarsal bones (9). TNF is also known to trigger the production of other pro-inflammatory cytokines, such as IL6 and IL-1beta (10), suggesting that TNF is a master player in the inflammatory process. Whether TNF alone may impair chondrogenesis in the human growth plate has so far not been studied.

Humanin, a 24-amino acid peptide first identified in surviving neurons from an Alzheimer disease patient, has been found to be a potent neurosurvival and anti-apoptotic factor (11). Humanin is also expressed in growth plate cartilage where it was shown to protect chondrocytes from undergoing undesired apoptosis (12). Interestingly, humanin itself was also found to exert anti-inflammatory effects (12). Recently, it was reported that humanin can improve metabolic health and increase lifespan in mice (13).

To identify any association between chronic inflammation and systemic humanin regulation, we measured humanin levels in serum samples obtained from IBD patients with known poor bone health. By applying a unique organ culture system of human growth plate tissues from children, we also investigated the local effects of IBD serum and TNF alone on local humanin expression and chondrogenesis within the growth plate.

## Materials and methods

### Serum collection from IBD patients and controls

We used previously collected serum samples from IBD children (boys and girls, median age 14.9 years) with known history of low bone mineral density and decreased height Z score, and gender-matched healthy children which served as controls (2).

### Serum humanin ELISA

Circulating humanin levels were measured in the serum of IBD patients and healthy controls by an ELISA kit (CSB-EL015084HU; CUSABIO, Houston, USA) according to the manufacturer’s instruction. We used the serum samples obtained from 40 IBD patients and 40 age matched healthy controls to measure humanin levels. Briefly, standards, or 10-fold diluted serum samples were added to the appropriate wells and incubated for 2 hours at 37°C. After removing the liquid of each well, 100 μl of Biotin-antibody was added and then incubated for 1 hour at 37°C. After washing, 100 μl of HRP-avidin was added to each well and incubated for 1 hour at 37°C followed by five times washing. Then, 90 μl of TMB Substrate was added to each well and incubated 25 minutes at 37°C followed by adding 50 μl Stop Solution. The absorbance was read at 450 nm on a microplate reader.

### Collection and culture of human growth plate tissues

Human growth plate tissues were collected during epiphysiodesis surgeries performed at Karolinska University Hospital. After informed consent, human growth plate samples were obtained from 2 children (1 boy, 1 girl) diagnosed with constitutional tall stature. Growth plate biopsies from the distal femur or proximal tibia were harvested by using a biopsy needle (8 gauge; Gallini Medical Products and Services, Modena, Italy) as earlier described by us (14). The biopsies were collected in DMEM, high glucose (21063-029; Thermo Fisher Scientific, Waltham, Massachusetts, USA) with 10 μg/ml gentamicin (11530506; Thermo Fisher Scientific, Waltham, Massachusetts, USA) and then kept on ice while being transported from the operating room to the laboratory for culture. Under aseptic conditions, the human growth plate biopsies were cut into 1-2 mm slices and each slice was then individually cultured in a 24-well plate with 0.5 ml culture media per well. The culture media was DMEM, high glucose (21063-029; Thermo Fisher Scientific, Waltham, Massachusetts, USA) supplemented with of 0.2% bovine serum albumin, 1 mM β-Glycerophosphate and 0.05 mg/ml ascorbic acid as described earlier (14). The human growth plate biopsies were subsequently treated and cultured in media with 10% serum, or 30 ng/ml TNF (510-RT-010; Bio-Techne, Minneapolis, Minnesota, USA) combined or not combined with 1 μM HNG for 48 hours, in a 5% CO_2_ incubator. Thereafter, the growth plates were fixed for 24 hours in 4% paraformaldehyde (HL96753.1000; Histolab, Askim, Sweden) followed by decalcification in EDTA buffer for 24 hours before dehydration and paraffin embedding.

### Immunohistochemistry and quantification

To analyze protein expression in the growth plate, immunohistochemistry was performed in serial sections of human growth plates as earlier described (12). Briefly, the sections were first deparaffinized and rehydrated. Antigen retrieval was then performed in sodium citrate buffer (10 mM pH 6.0) for 10 min at 75°C followed by washing with distilled water. Thereafter, sections were quenched with 3% hydrogen peroxide (1072090250; Burlington, Massachusetts, USA) in methanol (1060092511; Burlington, Massachusetts, USA) for 10 minutes and blocked with 2% goat serum for 1 hour. Next, slides were incubated with anti-humanin antibody (NB100-56877; Novus Biologicals, Littleton, Colorado, USA), anti-proliferating cell nuclear antigen (PCNA) antibody (ab-18197; Abcam, Cambridge, United Kingdom), anti-SOX9 antibody (ab-5355; Sigma-Aldrich, Burlington, MA, USA), and anti-TRAF2 antibody (NB100-56173SS; Novus Biologicals, Littleton, Colorado, USA) overnight at 4°C, 1:300 diluted for all antibodies. After primary antibody incubation, sections were washed with PBS with 0.05% Tween 20 (P1379; Sigma-Aldrich, Burlington, MA, USA) for 15 minutes followed by incubation with secondary antibody (1:300; BA-9500 Vector Laboratories; 1:500, ab 97049 Abcam) for 1 hour at room temperature. Sections were then incubated with an avidin-peroxidase complex (Vectastain ABC-kit PK-6100) and visualized with 3,3’ diaminobenzidine (DAB) (Dako K3468) development for 1-3 minutes. Finally, the slides were counterstained with Alcian Blue for 5 minutes and dehydrated. Image J software (NIH) was used to quantify the percentages of positive stained cells per mm² in the growth plates. Each growth plate slice was regarded as one observation.

### Bone growth analysis in cultured bones

The metatarsal bones were dissected from the hind paws of 17-18 days old fetal Sprague-Dawley rats (E17/18) as previously described (9). Only the middle three metatarsal bones were collected. Thereafter, the bones were cultured in 24-well plates with 0.5 ml/well of culture media. The culture media used was MEM (31095029; Thermo Fisher Scientific, Waltham, Massachusetts, USA) supplemented with 50 μg/ml ascorbic acid (A5960-100G; Sigma-Aldrich, Burlington, MA, USA), 1 mM sodium glycerophosphate (G9422-10G; Sigma-Aldrich, Burlington, MA, USA), 0.2% bovine serum albumin (A8806-5G; Sigma-Aldrich, Burlington, MA, USA), and 20 μg/ml gentamicin. The metatarsals were treated with 100 ng/ml TNF (510-RT-010; Bio-Techne, Minneapolis, Minnesota, USA), 1 μM humanin analog HNG, or both for 12 days at 37°C with 5% CO_2_. Images of the bones were captured on days 0, 2, 5, 7, 9, and 12 of culture using a Hamamatsu C4742–95 digital camera mounted on a Nikon SMZ-U microscope. The bone length was measured with the Infinity Analyze software (Lumenera Corporation).

### Cell culture

The human chondrocytic cell line, HCS-2/8 was cultured as described previously (15). Briefly, the cells were maintained in DMEM/F-12 (11320033; Thermo Fisher Scientific, Waltham, Massachusetts, USA) supplemented with 20% fetal bovine serum (FBS) and 20 μg/ml gentamycin at 37°C with 5% CO_2_. The cells were sub-cultured every week and the culture medium was changed every 2-3 days. For quantitative realtime PCR and Western blot analysis, the cells were seeded in 6-well cell culture plates in DMEM/F12 medium containing 20% FBS. When cells were approximately 80% confluent, they were washed with 1× PBS and the medium was changed to test medium (DEME/12 without FBS) with TNF (510-RT-010; Bio-Techne, Minneapolis, Minnesota, USA) for 72 hours.

### RNA extraction and quantitative real-time PCR

Total RNA was extracted from harvested HCS-2/8 cells using TRIzol reagent (15596026; Thermo Fisher Scientific, Waltham, Massachusetts, USA) according to the manufacturer’s instruction. iScript™ cDNA Synthesis Kit (1708890; Bio-Rad, Hercules, California, USA) was used for the reverse transcription of total RNA into cDNA. SsoAdvanced Universal SYBR Green Supermix (1725271; Bio-Rad, Hercules, California, USA) was used for quantitative real-time PCR (qPCR) reactions. The primers of PCNA (Assay ID: qHsaCID0012792), humanin (Assay ID: qHsaCED0019576), SOX9 (Assay ID: qHsaCED0021217), and Beta-actin (Assay ID: qHsaCED0036269) were purchased from Bio-Rad. qPCR reactions were performed with Bio-Rad 96 CFX RT-PCR System. Beta-actin was used as an internal reference gene to normalize the target genes. Relative levels of target genes were calculated using the ΔΔCT method.

### Western blot analysis

Western blot analysis was performed as previously described (16). The antibody against SOX9 (1:500; ab-5355) was purchased from Sigma-Aldrich, Burlington, MA, USA). The antibody against PCNA (1:1000; ab-18197) was purchased from Abcam (Cambridge, United Kingdom). Anti-GAPDH antibody (1:2000; 10494-1-AP) and Goat anti-Rabbit IgG (H+L) Secondary Antibody (1:3000; 65-6120) were purchased from Thermo Fisher Scientific (Waltham, Massachusetts, USA). Image J software (NIH) was used to quantify the images obtained from Western blots.

### Immunofluorescence

HCS-2/8 cells were seeded on Falcon 8-well Culture Slide (354108; Corning, USA), treated with TNF (510-RT-010; Bio-Techne, Minneapolis, Minnesota, USA) at 100 ng/ml concentration for 72 hours. Thereafter, the cells were washed with PBS and fixed in pure methanol (1060092511; Burlington, Massachusetts, USA) for 10 minutes at −20°C. After washing with PBS, fixed cells were blocked in 5% bovine serum albumin (A8806-5G; Sigma-Aldrich, Burlington, MA, USA) for 1 h at room temperature and incubated overnight at 4°C with anti-humanin antibody (1:500; NB100-56877; Novus Biologicals, Littleton, Colorado, USA). After primary antibody incubation, cells were washed with PBS with 0.05% Tween 20 (P1379; Sigma-Aldrich, Burlington, MA, USA) for 15 minutes followed by incubation with Cy3 AffiniPure F(ab’)_2_ Fragment Donkey Anti-Rabbit IgG (H+L) secondary antibody (1:500; 711-166-152; Jackson ImmunoResearch, United Kingdom) for 1 hour at room temperature. Nuclei were stained with Hoechst 33342 (1:500; H3570; Thermo Fisher Scientific, Waltham, Massachusetts, USA) together with secondary antibody incubation. The coverslips were mounted in Prolong Gold antifade reagent (P36930; Thermo Fisher Scientific, Waltham, Massachusetts, USA). Images were captured on an LSM 700 confocal microscope (ZEISS, Jena, Germany). Quantification of the fluorescence intensity in each image was performed using ZEN Microscopy Software (ZEISS, Jena, Germany).

### Statistical analysis

All statistical analyzes and receiver operating characteristics (ROC) analysis were performed in GraphPad Prism. 2-tailed unpaired t test and Wilcoxon rank-sum test were used to evaluate statistical significance between 2 groups. 2-way ANOVA was used for the analysis in the fetal metatarsal experiment. All data are shown as mean ± SE, and a *p*-value less than 0.05 was considered significant.

## Results

### Decreased humanin levels in serum from IBD patients

**Figure 1:**
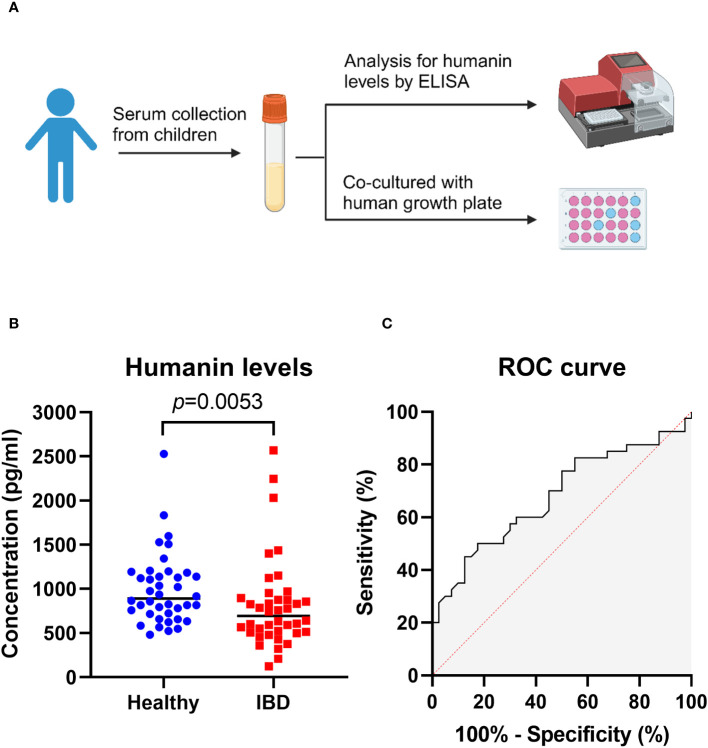
Humanin levels were decreased in serum of IBD patients. **(A)** Graphical illustration (created with BioRender.com) of the experimental overview, showing serum samples were obtained from children for ELISA analysis and human growth plate co-culture. **(B)** Humanin levels analyzed by using ELISA in IBD patients (n=40) and healthy controls (n=40). Wilcoxon rank-sum test was used to analyze differences between groups. **(C)** Receiver operating characteristic (ROC) curve for circulating humanin in IBD patients (n=40) and healthy controls (n=40).

We first measured humanin levels in serum samples obtained from 40 patients with IBD (median age 14.9 years) and 40 age-matched healthy controls. Humanin levels were found to be significantly decreased in IBD patients (*p*=0.0053 vs controls) (**Figures 1A, B**) suggesting a link between inflammation and systemic humanin regulation. To investigate the sensitivity and specificity of circulating humanin in the serum samples, a receiver operating characteristics (ROC) curve was constructed (**Figure 1C**). The ROC curve analysis revealed that the area under the curve (AUC) value for circulating humanin is 0.68 (95% confidence interval: 0.56 to 0.80).

### Decreased humanin expression in human growth plate cartilage exposed to IBD serum

**Figure 2:**
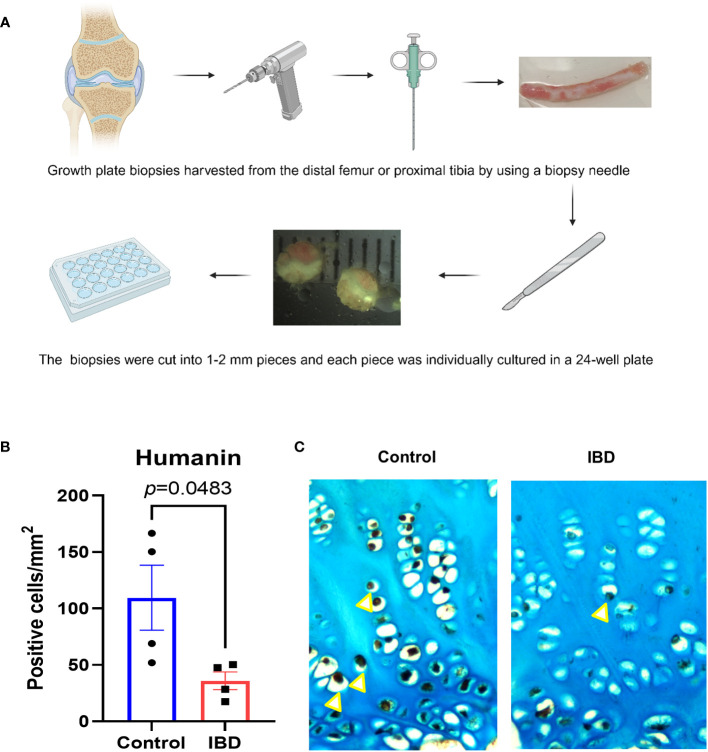
Humanin levels were decreased in human growth plate exposed to IBD serum. **(A)** Graphical illustration (created with BioRender.com) of the methods using to collect and culture human growth plate biopsies obtained from children undergoing epiphysiodesis surgeries. **(B, C)** Quantitative analysis of humanin staining (yellow arrows), quantified as number of positive cells per mm² (n=4 pieces of biopsies, from 1 patient). Error bars indicate mean ± SE. Students t-test was used to analyze differences between groups.

To test if serum obtained from children with IBD may locally affect humanin expression in the growth plate, tissue specimens obtained from a human growth plate were cultured with serum from IBD patients and healthy controls for 48 hours (**Figure 2A**). Immunohistochemistry analysis showed that humanin levels were significantly decreased in growth plate specimens exposed to serum obtained from IBD patients when compared to healthy controls (*p*=0.0483) (**Figures 2B, C** and **Supplementary Figure 1A**).

### TNF suppressed humanin expression in cultured human growth plate cartilage and chondrocytes

**Figure 3:**
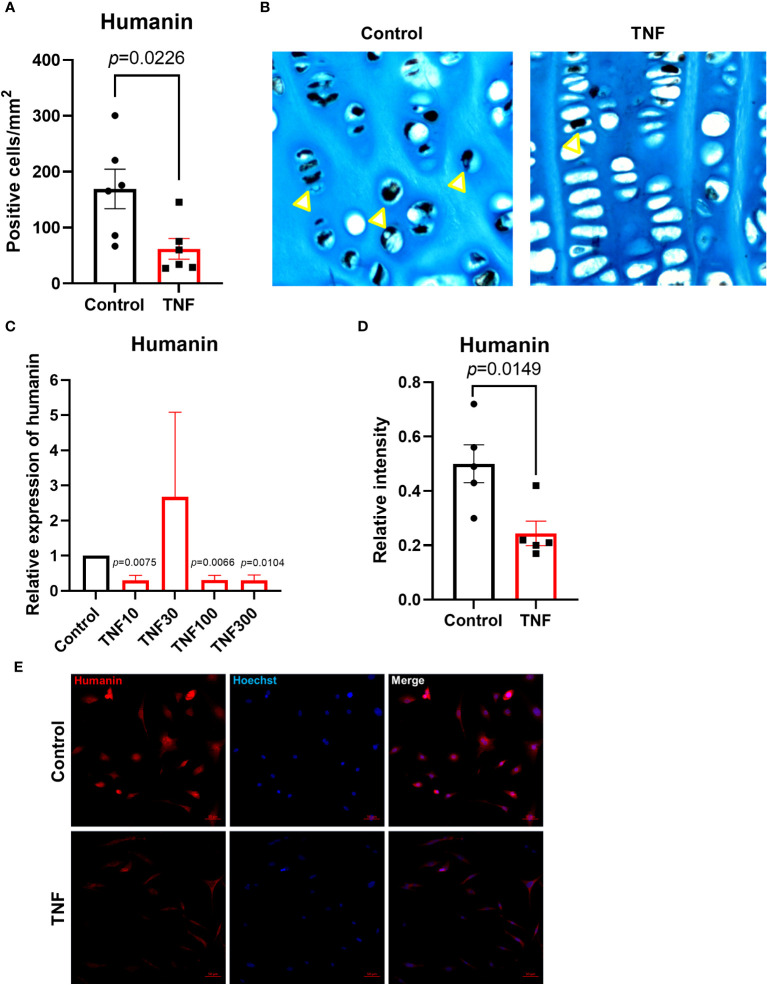
TNF suppressed humanin expression in human growth plate tissue specimens (n=6) obtained from 2 children or human chondrocytes. **(A, B)** Quantitative analysis of humanin staining (yellow arrows), calculated as number of positive cells per mm². **(C)** Relative expression of humanin assessed by qPCR in HCS-2/8 cell line treated for 72 hours with TNF at 10, 30,100, 300 ng/ml concentrations (n=3-4). **(D)** Quantification of humanin expression by immunofluorescence in HCS-2/8 cells treated with TNF (100 ng/ml). **(E)** Representative images of humanin staining (red) in HCS-2/8 cells treated with TNF and untreated controls. Error bars indicate mean ± SE. Students t-test was used to analyze differences between groups.

**Figure 4:**
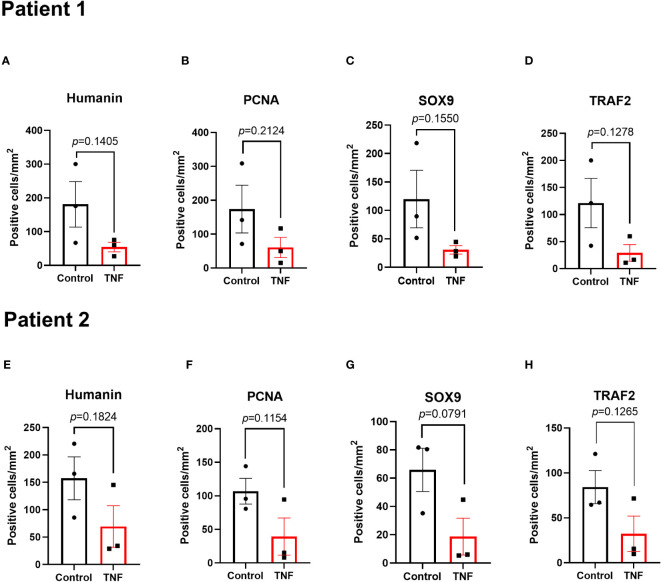
Local effects of TNF on humanin, PCNA, SOX9 and TRAF2 expressions in growth plates tissue specimens (n=3) in patient 1 and patient 2 analyzed separately. Quantitative analysis of **(A, E)** humanin staining, **(B, F)** PCNA staining, **(C, G)** SOX9 staining, and **(D, H)** TRAF2 staining. Error bars indicate mean ± SE. Students t-test was used to analyze differences between groups.

To test if the important pro-inflammatory cytokine TNF may have a direct suppressive effect on humanin expression in growth plate cartilage, growth plate tissue specimens obtained from 2 different children were cultured with TNF (30 ng/ml) or control medium for 48 hours. Our data showed that TNF significantly suppressed humanin expression within the human growth plate cartilage (*p*=0.0226) (**Figures 3A, B** and **Supplementary Figure 1B**). A similar, although not significant, trend was found when data from the 2 patients were analyzed separately (**Figures 4A, E**).

To investigate the dose-response effect of TNF on humanin expression in human chondrocytes, we treated the HCS-2/8 chondrocytic cell line with TNF at 10, 30, 100, and 300 ng/ml for 72 hours. The qPCR results showed that humanin was significantly suppressed by TNF at 10, 100, and 300 ng/ml vs control (*p*=0.0075, 0.0066 and 0.0104, respectively) (**Figure 3C**). To further validate this data, we performed immunofluorescence and found that humanin was significantly suppressed in the HCS-2/8 chondrocytic cell line treated with TNF (*p*=0.0149) (**Figures 3D, E**).

### TNF suppressed SOX9 and PCNA in cultured human growth plate cartilage and chondrocytes

**Figure 5:**
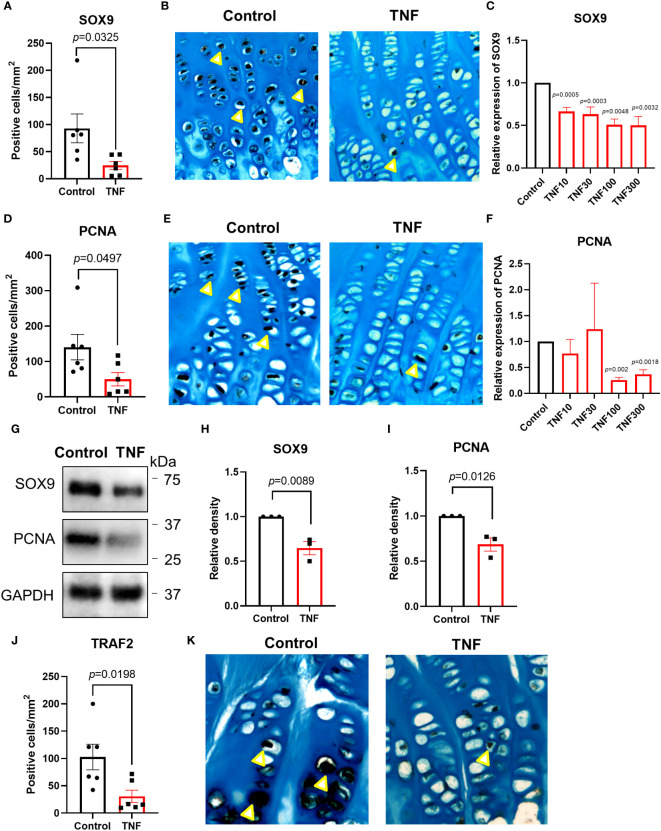
TNF suppressed SOX9, PCNA and TRAF2 expressions in human growth plate tissue specimens (n=6) obtained from 2 children or human chondrocytes. **(A, B)** Quantitative analysis of SOX9 staining (yellow arrows), calculated as number of positive cells per mm². **(C)** Relative expression of SOX9 assessed by qPCR in HCS-2/8 cells treated for 72 hours with TNF at 10, 30, 100, 300 ng/ml concentrations (n=3). **(D, E)** Quantitative analysis of PCNA staining (yellow arrows), calculated as number of positive cells per mm². **(F)** Relative expression of PCNA assessed by qPCR in HCS-2/8 cells treated for 72 hours with TNF at 10, 30, 100, 300 ng/ml concentrations (n=3). **(G)** Western blot analysis of SOX9 and PCNA expressions in HCS-2/8 cells treated with TNF (100 ng/ml). **(H, I)** Quantification of SOX9 and PCNA expressions by three independent Western blot experiments. **(J, K)** Quantitative analysis of TRAF2 staining (yellow arrows), calculated as number of positive cells per mm². Error bars indicate mean ± SE. Students t-test was used to analyze differences between groups.

To investigate if TNF alone may suppress chondrogenesis in the human growth plate, tissue specimens were further analyzed for expressions of SOX9 and PCNA. When treated with TNF alone, SOX9 expression was decreased by 73.4% vs control (*p*=0.0325) (**Figures 5A, B** and **Supplementary Figure 2A**). Similarly, PCNA, a marker of cell proliferation, was suppressed by TNF (*p*=0.0497) (**Figures 5D, E**, **Supplementary Figure 2B**). When analyzing data from patient 1 and patient 2 individually, a similar trend was noticed in both patients (**Figures 4B, F, C, G**).

We next performed dose-response studies in the HCS-2/8 cell line with qPCR and observed that TNF significantly decreased SOX9 gene expression at 10, 30, 100, and 300 ng/ml vs control (*p*=0.0005, 0.0003, 0.0048 and 0.0032, respectively) (**Figure 5C**). Similarly, PCNA expression was suppressed by TNF at 100 and 300 ng/ml vs control (*p*=0.002 and 0.0018, respectively) (**Figure 5F**). Western blot analysis also showed that SOX9 and PCNA were significantly decreased in the HCS-2/8 chondrocytic cell line treated with TNF (*p*=0.0089 and 0.0126, respectively) (**Figures 5G–I**).

### TNF suppressed TRAF2 in cultured human growth plate cartilage

Since TRAF2 plays an important role in TNF-induced inflammatory signaling, TRAF2 expression was measured in human growth plate tissue specimens treated with TNF. Our data showed that TNF suppressed TRAF2 expression by 70.1% vs control (*p*=0.0198) (**Figures 5J, K** and **Supplementary Figure 2C**). When analyzing the two patients separately, a similar trend was noticed in both patients (**Figures 4D, H**).

### Treatment with humanin analog prevents TNF-induced growth impairment in cultured bones

**Figure 6:**
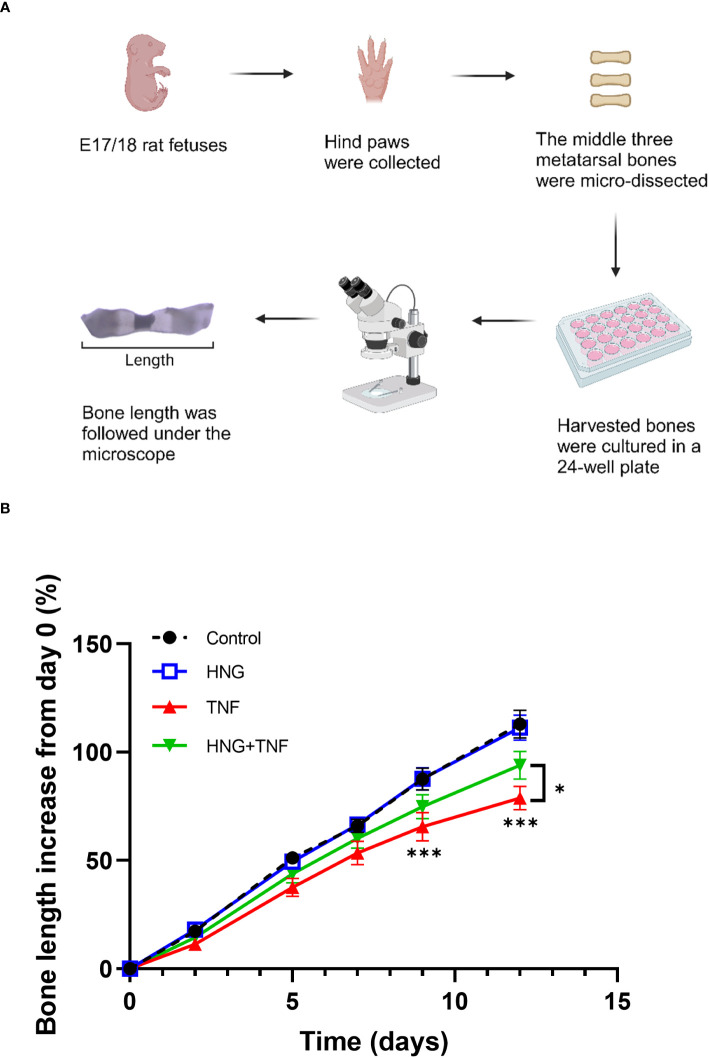
Treatment with humanin analog HNG prevents TNF-induced growth impairment in cultured bones. **(A)** Microscopic image of *ex vivo* cultured fetal rat metatarsal bones. **(B)** *Ex vivo* cultured fetal rat metatarsal bones treated with HNG (1 µM), TNF (100 ng/ml) or in combination for 12 days (n=12). Bone length was measured on day 0, 2, 5, 7, 9 and 12. Error bars indicate mean ± SE. 2-way ANOVA was used to analyze differences between groups. *p < 0.05 (TNF versus HNG+TNF), ***p < 0.001 (Control versus TNF).

To investigate whether humanin could be a therapeutic target to prevent bone growth impairment caused by chronic inflammation, cultured rat metatarsal bones were treated with TNF or the humanin analog HNG, or in combination for 12 days. We observed that TNF alone impaired bone growth (*p*<0.0001 vs control), whereas co-treatment with HNG rescued bone growth (*p*= 0.0436 vs TNF) (**Figures 6A, B**), confirming the therapeutic effect of humanin.

## Discussion

We here report a novel direct link between chronic inflammation and humanin regulation in children. Such link is supported by our findings of decreased serum humanin levels in IBD patients and suppressed humanin expression in *ex vivo* cultured human growth plate tissues exposed to IBD serum or TNF alone.

To our knowledge, there is no previous evidence supporting any link between chronic inflammation and humanin regulation. To address this gap of knowledge, we measured humanin levels in serum samples from 40 IBD patients and 40 age and gender matched healthy controls. Our data showed that humanin levels were significantly decreased in children with IBD. Humanin is a mitochondrial-derived polypeptide, first identified in survived neuronal cells from Alzheimer disease patients (11). Under physiological conditions, humanin is produced by various tissues such as skeletal muscle, brain, and liver (17–19). After production, it is circulated in the blood and plays a protective role in targeted cells (11). In the cytoplasm, humanin is known to protect cells from apoptosis by interacting with pro-apoptotic proteins (20). Similarly, humanin can also interact with extracellular receptors such as G protein-coupled formylpeptide receptor-like-1 to exert its cell-protective effects (21). The kinetic half-life of humanin is 30 minutes in the plasma of mice and greater than 4 hours in rats (22). Previous studies have reported that in multiple species including human, circulating humanin levels gradually decrease with age (13). In addition, systemic humanin levels are suppressed in patients with Alzheimer’s disease and coronary endothelial dysfunction (13, 23). The mechanism of how humanin is decreased by chronic inflammation is still unclear. However, it has been reported that when mitochondrial DNA copy number decreased, humanin levels were also decreased (13). Interestingly, inflammation is known to decrease the mitochondrial DNA copy number (24). These observations may explain a possible link between inflammation and humanin regulation.

We have previously reported that humanin is expressed in the growth plate and that humanin has the capacity to prevent glucocorticoid-induced bone growth impairment (12). Furthermore, glucocorticoids have been reported to suppress humanin expression in the growth plate (12). However, so far it has been unknown if chronic inflammation *per se* also may suppress endogenous humanin produced within the growth plate. Considering the protective roles of humanin in different diseases reported earlier (17, 25, 26), we hypothesized that humanin might be a novel biomarker of poor bone health in patients with chronic inflammation. To investigate this, growth plate cartilage was collected from children with constitutional tall stature undergoing epiphyseal surgery performed to limit their further bone growth. A unique human growth plate culture system (14) was then applied which allowed tissue specimens to be exposed to serum obtained from IBD patients or healthy controls. Interestingly, we found suppressed humanin expression in growth plates exposed to IBD serum suggesting a direct link between chronic inflammation and local humanin regulation within the growth plates.

In an attempt to clarify a possible underlying mechanism behind inflammation-induced humanin suppression, we focused on TNF, a key player in chronic inflammation triggering the release of other pro-inflammatory cytokines such as IL-6 and IL-1beta (10). Although TNF overexpression was recently reported by us to suppress chondrocyte proliferation and chondrogenesis within the mouse growth plate, any such effects have not yet been reported in humans (27). In this study, we observed a strong inhibition of humanin expression in human growth plates exposed to TNF for 48 hours. Further studies showed that the proliferation marker PCNA and the important controller of chondrocyte proliferation, SOX9, were both markedly decreased in growth plates exposed to TNF. Heterozygous mutations of SOX9 have been described in patients with campomelic dysplasia, a skeletal dysplasia characterized by bowed long bones and defects in cartilage formation (28). Furthermore, several animal studies have demonstrated that intact SOX9 is essential for proliferative chondrocyte columns and keeping the growth plates open (29, 30). Consequently, our data showing suppressed SOX9 in growth plates exposed to TNF may explain why this pro-inflammatory cytokine exerts growth suppressive effects. Interestingly, we also found that TNF Receptor Associated Factor 2 (TRAF2) was suppressed in human growth plates exposed to TNF. These novel findings suggest a direct link between chronic inflammation and humanin regulation, both systemically and locally within human growth plates.

To validate the data collected from human growth plates, we performed dose-response studies using the human chondrocytic cell line HCS-2/8. The rationale to choose this cell line was that HCS-2/8 cells resemble primary human chondrocytes and have been widely used to study chondrocyte proliferation and differentiation since established (31). As expected, the expressions of humanin, SOX9, and PCNA in HCS-2/8 cells were suppressed when exposed to TNF. These findings are in line with the data collected in cultured human growth plates.

Since humanin was suppressed under chronic inflammation, we next investigated whether exogenous treatment with humanin can rescue from inflammation-induced bone growth impairment. In an *ex vivo* model of cultured bones where direct effects on bone growth can be monitored (32), we found that humanin can effectively protect from TNF-induced bone growth impairment. This observation further implies a potential link between humanin and poor bone growth under chronic inflammation. The clinical significance of our finding is underscored by the fact that existing treatments are associated with various side effects. Biological drugs such as etanercept (TNF-inhibitor) and anakinra (IL-1 receptor antagonist) has been reported to cause infections and injection-site reactions (33). Similarly, the long-term safety of recombinant human growth hormone is associated with increased mortality in certain patient groups and the cost of this therapy is also very high (34). Therefore, new treatment strategies are highly desired to prevent bone growth impairment caused by chronic inflammation. Moreover, glucocorticoids (GCs) are also widely used in the management of IBD and several attempts have been made to reduce the toxicity of GCs (35–37). Interestingly, it has been reported that the combination of the humanin analog HNG with a GC does not interfere with the desired anti-inflammatory effects of the GC (12, 38). Thus, it would be of great clinical interest to further expand the scope of the present study regarding the therapeutic effects of humanin.

There are several limitations of this study. Firstly, growth plate cartilage was obtained from 2 children due to the rarity of these samples. However, as these biopsies could been sectioned into several slices being cultured and treated individually the number of replicates could be increased. A second limitation was that we could not perform dose-response studies, again due to the scarcity of human growth plate tissues. Thirdly, the serum samples from IBD patients in our study were obtained only at one time point, therefore, more detailed studies are required to reveal whether circulating humanin could potentially act as a biomarker of poor bone health in children with IBD. The last limitation was that we did not explore the role of other pro-inflammatory cytokines than TNF and it is therefore possible that other pathways may also be involved in mediating the local effects of chronic inflammation on humanin regulation.

## Conclusion

We report that systemic humanin levels are decreased in IBD children with poor bone health. Mechanistic studies in *ex vivo* cultured human growth plate cartilage and human chondrocytes showed that serum from IBD patients or TNF alone suppressed endogenous humanin expression. Furthermore, treatment with the humanin analog HNG prevented growth retardation caused by TNF in cultured bones. Altogether, our study provides evidence of a link between chronic inflammation, bone health and humanin regulation, which is a novel finding of potential clinical significance.

## Data availability statement

The raw data supporting the conclusions of this article will be made available by the authors, without undue reservation.

## Ethics statement

The studies involving humans were approved by Helsingin ja Uudenmaan sairaanhoitopiiri, Finland, and Karolinska Institutet Research Ethics Committee North at the Karolinska University Hospital, Stockholm, Sweden. The studies were conducted in accordance with the local legislation and institutional requirements. Written informed consent for participation in this study was provided by the participants’ legal guardians/next of kin. The animal study was approved by the local ethical committee (Stockholm North Animal Ethics Committee. The study was conducted in accordance with the local legislation and institutional requirements.

## Author contributions

YZ participated in study design, experimental work, statistics, data analysis, manuscript writing. OM provided samples, reviewed manuscript. SL provided samples, reviewed manuscript. VF participated in experimental work, data analysis and manuscript writing. LS participated in study design, statistics, data analysis, manuscript writing, study concept, manuscript revision. FZ participated in study design, experimental work, statistics, data analysis, manuscript writing. All authors contributed to the article and approved the submitted version.

## Supplementary material

Supplementary Figure 1 Humanin levels were decreased in human growth plate tissue specimens exposed to IBD serum and TNF. **(A)** Representative images of immunohistochemistry for humanin (dark brown staining) in human growth plate tissue specimens exposed to serum from IBD patients and healthy controls. **(B)** Representative images of immunohistochemistry for humanin (dark brown staining) in human growth plate tissue specimens treated with TNF and untreated control. 10x magnification.

Supplementary Figure 2 TNF suppressed SOX9, PCNA and TRAF2 expressions in human growth plate tissue specimens (n=6) obtained from 2 children or human chondrocytes. **(A–C)** Representative images of immunohistochemistry for SOX9, PCNA and TRAF2 (dark brown staining) in human growth plate tissue specimens treated with TNF and untreated controls. 10x magnification.
