# Supplementary figures and images for: A novel link between chronic inflammation and humanin regulation in children
Source: Front Endocrinol (Lausanne). 2024 Jan 23;14:1142310. doi: 10.3389/fendo.2023.1142310 (PMC10844658; doi:10.3389/fendo.2023.1142310)

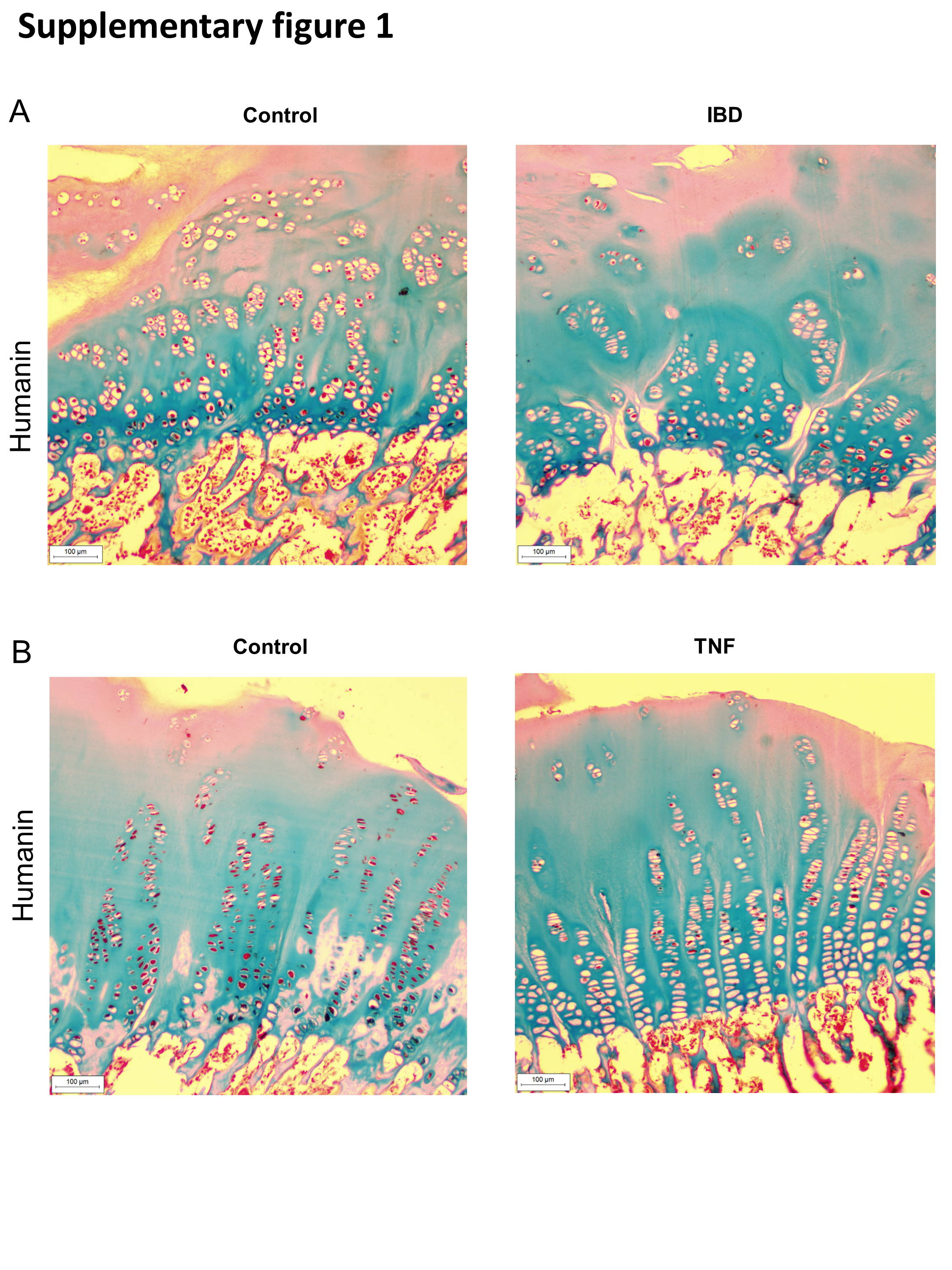

Supplement: Supplementary Figure 1 — Humanin levels were decreased in human growth plate tissue specimens exposed to IBD serum and TNF. (A) Representative images of immunohistochemistry for humanin (dark brown staining) in human growth plate tissue specimens exposed to serum from IBD patients and healthy controls. (B) Representative images of immunohistochemistry for humanin (dark brown staining) in human growth plate tissue specimens treated with TNF and untreated control. 10x magnification. [file Image_1.tif]

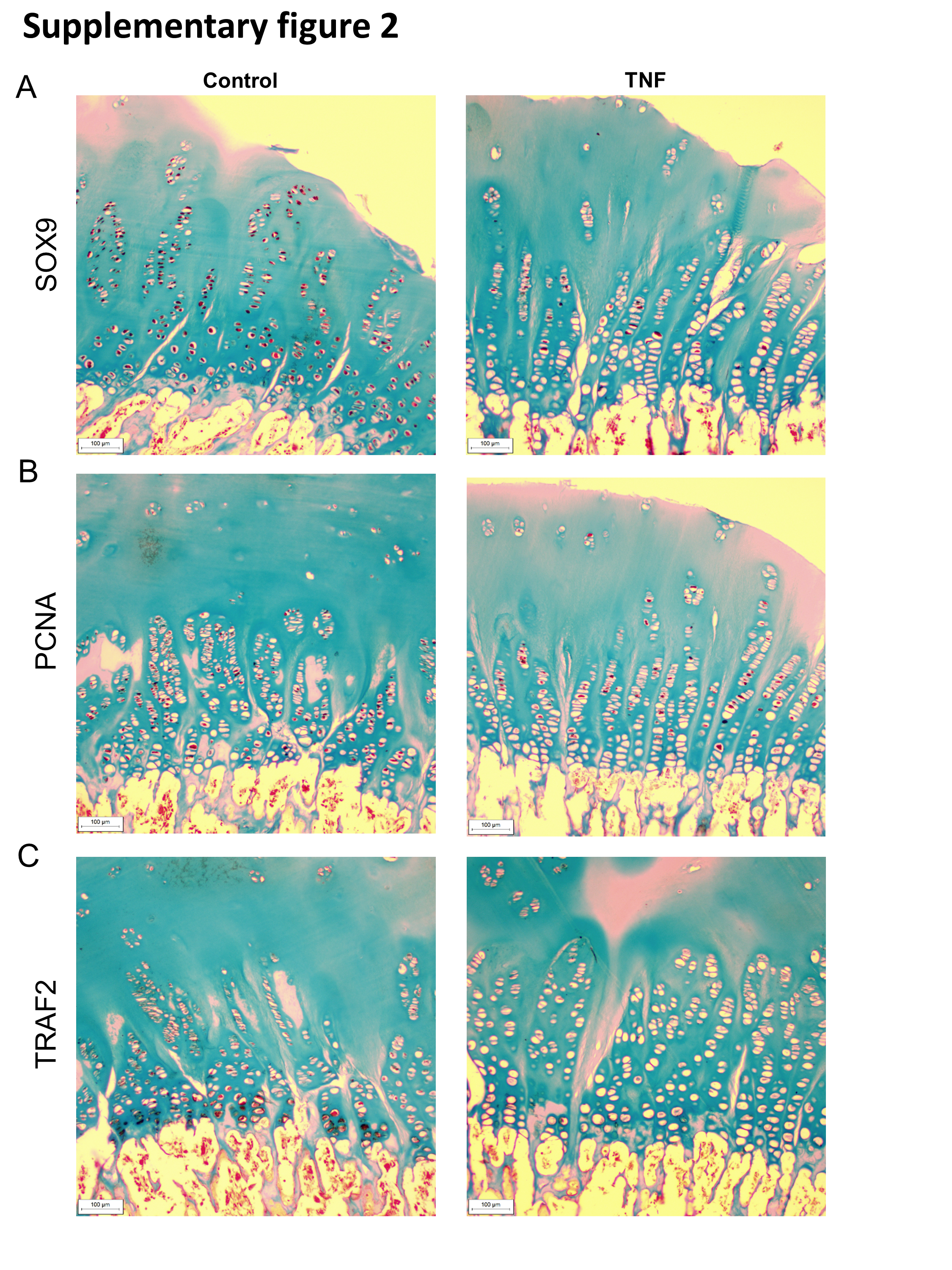

Supplement: Supplementary Figure 2 — TNF suppressed SOX9, PCNA and TRAF2 expressions in human growth plate tissue specimens (n=6) obtained from 2 children or human chondrocytes. (A–C) Representative images of immunohistochemistry for SOX9, PCNA and TRAF2 (dark brown staining) in human growth plate tissue specimens treated with TNF and untreated controls. 10x magnification. [file Image_2.tif]
